# Responses of functional brain networks in micro-expressions: An EEG study

**DOI:** 10.3389/fpsyg.2022.996905

**Published:** 2022-10-28

**Authors:** Xingcong Zhao, Jiejia Chen, Tong Chen, Shiyuan Wang, Ying Liu, Xiaomei Zeng, Guangyuan Liu

**Affiliations:** ^1^School of Electronic and Information Engineering, Southwest University, Chongqing, China; ^2^Key Laboratory of Cognition and Personality, Ministry of Education, Southwest University, Chongqing, China; ^3^School of Music, Southwest University, Chongqing, China

**Keywords:** micro-expressions, inhibitory control, electroencephalography, brain connectivity, emotion

## Abstract

Micro-expressions (MEs) can reflect an individual’s subjective emotions and true mental state, and they are widely used in the fields of mental health, justice, law enforcement, intelligence, and security. However, one of the major challenges of working with MEs is that their neural mechanism is not entirely understood. To the best of our knowledge, the present study is the first to use electroencephalography (EEG) to investigate the reorganizations of functional brain networks involved in MEs. We aimed to reveal the underlying neural mechanisms that can provide electrophysiological indicators for ME recognition. A real-time supervision and emotional expression suppression experimental paradigm was designed to collect video and EEG data of MEs and no expressions (NEs) of 70 participants expressing positive emotions. Based on the graph theory, we analyzed the efficiency of functional brain network at the scalp level on both macro and micro scales. The results revealed that in the presence of MEs compared with NEs, the participants exhibited higher global efficiency and nodal efficiency in the frontal, occipital, and temporal regions. Additionally, using the random forest algorithm to select a subset of functional connectivity features as input, the support vector machine classifier achieved a classification accuracy for MEs and NEs of 0.81, with an area under the curve of 0.85. This finding demonstrates the possibility of using EEG to recognize MEs, with a wide range of application scenarios, such as persons wearing face masks or patients with expression disorders.

## Introduction

A micro-expression (ME) represents the facial leakage of a true emotion when an individual attempts to conceal that emotion; it provides a reliable indication of a person’s true intentions (Johnson et al., 1975). As instantaneous expressions, MEs are faint and difficult to recognize by the naked eye; however, they are believed to reflect a person’s true intent, especially one of a hostile nature (Dobson and Sherwood, 2010; Porter and ten Brinke, 2010; Porter et al., 2012). Therefore, ME research is important in several areas, such as mental health, justice, law enforcement, intelligence, and security. Currently, ME identification relies on image recognition technology focused on facial expression (Peng et al., 2017; Li et al., 2018; Ben et al., 2021). However, recording a person’s face is not always possible, for example when wearing a facemask or in low-visibility environments, or insufficient patients with expression disorders (Haenzi et al., 2014; Xu et al., 2022). In these circumstances, physiological measures, such as electroencephalography (EEG), represent a promising method that may address these issues, with a wide range of application scenarios. Therefore, this study explores for the first time the brain mechanisms of MEs from a neuroscience perspective and provides electrophysiological indicators for ME recognition.

The inhibition hypothesis proposed by Ekman (Malatesta, 1985) suggests that the occurrence of MEs involves both emotional arousal and voluntary cognitive control processes. For instance, when a person tries to suppress or conceal the expression of their true emotions in social situations, the pyramidal and extrapyramidal motor systems are activated simultaneously. When an emotion is triggered, the subcortical brain regions send an involuntary strong signal to the facial nerve. The individual then recruits their voluntary motor cortex to conceal this response, sending a signal to suppress their expression in a socially and culturally acceptable manner. These contrasting signals create a conflict over face control, and when the subcortical impulse prevails, the expression appears on the face as a fleeting ME before the voluntary motor system regains control of the facial muscles (Frank and Svetieva, 2015). Therefore, the duration of MEs is minimal, approximately 0.04–0.5 s, and they are challenging to see (Yan et al., 2013). In contrast, with millisecond temporal resolution, non-invasive and low risk, EEG has been widely used to study brain mechanisms of emotional arousal and cognitive control (Shahabi and Moghimi, 2016; Adelhofer et al., 2020).

Previous studies have shown that the interactions between multiple brain regions (e.g., areas controlling emotional arousal and cognition) can be accurately represented by functional connectivity and interactions between the corresponding brain networks (Misic and Sporns, 2016; Bartholomew et al., 2019; Cea-Canas et al., 2020). However, the results from functional connectivity research have not yet described the characteristics of the affective functional network as a whole, the connectome of the brain (Bullmore and Sporns, 2009). Graph theory metrics is an advanced research tool used to quantify the properties of all connections between a set of brain regions or nodes using the concept of efficiency, measuring how efficiently information is exchanged within the network (Bullmore and Sporns, 2009; Zhang et al., 2015). Therefore, a growing number of studies have used network modeling and graph theoretical analysis (GTA) to elucidate the complex relationships between brain regions (Bartholomew et al., 2019). For example, Kilic and Aydin (2022) found that GTA metrics, especially global efficiency, can effectively measure brain networks related to different discrete emotions and have a classification accuracy of approximately 80% (Kilic and Aydin, 2022). Zhang et al. (2015) found that the efficiency of the emotional functioning network was higher when participants observed emotional pictures than neutral ones, implying that local connections increase during the viewing of pictures with an affective meaning (Zhang et al., 2015).

Currently, the suppression-elicitation and the lying-leakage paradigms are the approaches typically used for ME elicitation (ten Brinke et al., 2012; Yan et al., 2014). The lying-leakage method (Ekman and Friesen, 1974; Frank and Ekman, 1997), although more ecologically valid, has the problem that the ME occurrence rate is quite low, and EEG studies require a certain number of occurrences before analysis. In contrast, the suppression-elicitation paradigm requires participants to maintain neutral facial expressions while watching a video eliciting strong emotions. Their performance is related to an experimental reward to increase the motivation to hide their true emotions in facial expressions (Yan et al., 2013, 2014). Video-induced high emotional intensity increases the occurrence of MEs; 109 MEs were detected among 1,000 facial expressions (approximately 11%). However, MEs mostly occur in interpersonal situations; thus, we added a real-time supervision module to the suppression-elicitation paradigm to improve the ecological validity of this method. Consequently, the participants and supervisors participated simultaneously in the experiments, providing a simulated social supervision situation. We named this improved paradigm real-time supervision and emotional expression suppression (SEES) experimental paradigm.

This study aimed to measure changes in brain networks during ME manifestation *via* the SEES paradigm and EEG techniques, enabling us to understand the neural mechanisms of MEs and provide electrophysiological indicators for ME recognition. Specifically, we combined a complex network analysis and machine learning algorithms to measure and classify the functional connectivity reorganizations of ME and NE signals in the presence of emotional stimuli. We hypothesized that MEs and NEs would be associated with different brain network patterns.

## Materials and methods

### Participants

A total of 70 self-reported right-handed participants were included in the present study. The participants were healthy and did not consume psychoactive substances. Participants at risk of depression (Beck Depression Inventory score >18) were excluded. Among these 70 participants, 65 exhibited at least one ME instance while watching the videos. Thus, the final sample comprised 65 participants (age range: 17–26 years; 23 men, 42 women). All participants provided written informed consent, and the study was approved by the ethics committee of Southwest University.

### Materials

We selected videos containing both visual and auditory emotional materials to elicit high-intensity emotions and physiological changes. We chose laughter as the target emotional expression. Highly amusing videos were used for SEES. Excerpts from Chinese comedy films and a variety of shows were used, as native culture factors may affect elicitation in emotion experiments. The criteria for selecting the video materials were as follows: (a) duration <3 min to avoid visual fatigue; (b) content that could be easily understood and did not require excessive thinking; and (c) content that elicited the expression of a single desired target emotion (i.e., urge to laugh). Based on these criteria, we manually selected 15 online videos as emotional materials. Twenty participants were asked to assess the valence of the videos, and they rated their emotional intensity on a 7-point Likert scale; 6 points and above was the criterion for selection. Finally, three videos were selected as elicitation material for the experiment and were carefully edited to ensure continuous emotional elicitation.

### Experimental design

We increased the psychological pressure of the classic ME paradigm to provide a situation with high emotional arousal and strong motivation to inhibit facial expressions. The participants and supervisors participated simultaneously in the experiments. Participants were seated approximately 1 m from a 23-inch screen; two cameras were placed behind the screen, a high-speed recording camera (90 frames/s) and a real-time surveillance camera on a tripod. The supervisor was seated approximately 1 meter to the left of the participant to observe the facial MEs through a monitor in real-time, and the participant was aware of the presence of the supervisor. Participants and supervisors were divided by a curtain to ensure that the movement of supervisors did not affect the participant’s attention. EEG signals were recorded from 128 active electrodes at a sampling rate of 1,024 Hz using a BioSemi Active system (BioSemi, Amsterdam, The Netherlands). A LabVIEW-based (National Instruments, Austin, TX, United States) synchronization system was then developed to synchronize accurately the EEG acquisition device and the high-speed camera. We ensured that the EEG signal was accurately synchronized with the acquisition of facial images using the same trigger simultaneously to generate time stamps on the camera recording and the BioSemi Active system.

The participants were instructed to remain relaxed and concentrate on the videos. They were also asked to maintain neutral facial expressions and avoid exaggerated expressions to conceal their true emotions. Additionally, they were told that their payment would be directly proportional to their performance. The supervisor assessed and judged the responses while the participants watched the three videos; if the supervisor could identify amused facial expressions, two yuan for each expression would have been subtracted from their payment. In contrast, if the supervisor did not observe any facial expressions, ten yuan would have been added as an extra reward. These strategies were employed to create a compelling stress situation and increase the motivation to hide genuine facial expressions.

### Facial expression identification

Complete MEs are difficult to elicit in the laboratory, and only partial facial expressions, such as movements from the lower or upper face, are typically observed (Porter et al., 2012; Yan et al., 2013). Nonetheless, partial MEs tend to be subtle manifestations of an underlying emotion, and observations of rapid facial expressions in various social situations indicate that MEs are more often partial than involving the full face (Porter et al., 2012). In this study, partial or full facial expressions with durations ≤500 ms were considered MEs. We first used action units (AUs) from a facial action-coding system (Yan et al., 2014) to detect MEs among participants at the same stimuli time points, based on a discriminative response map-fitting method (Asthana et al., 2013) tracking 66 facial landmarks of facial expressions (Liu et al., 2016). Two coders were involved in the analysis of the MEs (Yan et al., 2014). The procedure comprised the following three steps:

1. Counting the time points of all MEs among all participants. This step was used to detect all the time points of ME appearance throughout the video viewing. We determined the approximate time points of the onset, apex, and offset frames by playing the recording at 1/3 speed.
2. Frame-by-frame coding. This step was utilized to determine the ME onset, apex, and offset frames based on the time points previously selected. The first frame that showed activation of AU6, AU12, or both was considered the onset frame. The apex frame was defined as the one recording the entire expression with the maximum intensity. The offset frame was the last before the face returned to its original neutral expression (Hess and Kleck, 1990; Hoffmann, 2010; Yan et al., 2013). The coders examined the minute changes between adjacent frames surrounding the ME onset, apex, and offset repeatedly to properly identify these frames. The ME durations were then calculated.
3. Determining the time points. Based on the apex frame, we selected the time points when 20 or more participants showed MEs. We determined the global time points by averaging the times of the apex frames. Based on these reference points, 2-s blocks of the corresponding EEG signals were extracted for each participant to perform the analysis. Participants with MEs occurring within those time points were included in the ME group and those who maintained neutral expressions in the NE group.

### EEG preprocessing

**Figure 1:**
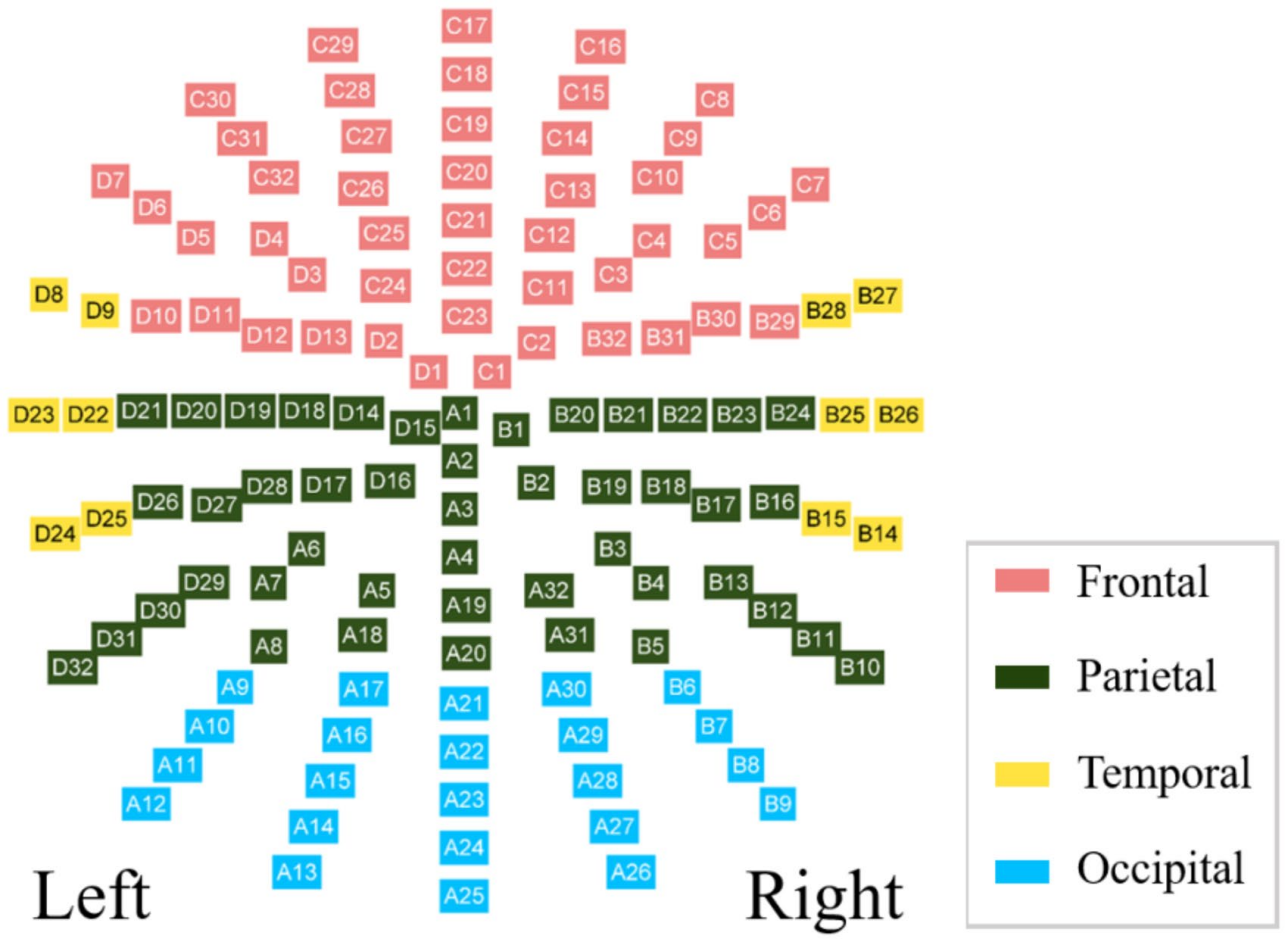
The positions of the EEG electrodes are divided into four regions.

The BioSemi Active electrodes recorded EEG signals referencing a common mode sense electrode as part of its feedback loop. An open-source toolbox was used in the MATLAB environment (MathWorks, Natick, MA, United States) using BESA research software (BESA GmbH, Gräfelfing, Germany). Drift and noise reduction were performed by applying a 0.5–60 Hz band-pass filter. The EEG signals were compared to the average reference to maximize the signal-to-noise ratio, and those contaminated by eye blinks and facial artifacts were corrected using the BESA research software. For further analyzes, we selected 128 electrodes and divided them into four regions: frontal, parietal, temporal, and occipital. The allocation scheme is shown in Figure 1.

### Brain network construction

Considering that the alpha, beta, and gamma bands are more sensitive to the emotion and cognition, we chose these three bands as the target bands for analysis. We constructed the brain networks in three classical frequency bands for each participant: alpha (8–12 Hz), beta (12–30 Hz), and gamma (30–60 Hz). The brain networks were constructed according to the following steps:

Step 1: Trimming the 2-s segments from the pre-processed EEG signals into 1-s blocks using the global field power (GFP) (Khanna et al., 2015). GFP is the root of the mean of the squared potential differences of all *K* channels (*V*_*i*_(*t*)) from the mean of the instantaneous potentials across channels (*V_mean_*(*t*)), according to the following formula:


$$GFP=\sqrt{\left(\overset{K}{\underset{i}{\sum }}{\left(V_{i}\left(t\right)-V_{mean}\left(t\right)\right)}^{2}\right)/K}$$

The 1-s block of data was selected with the maximum peak of GFP as the midpoint.

Step 2: Calculating the phase-locking value (PLV) matrix (Schmidt et al., 2014), which comprises the functional connectivity between all possible pairs of 128 nodes.

Step 3: Converting the PLV matrix into a brain network or a binary graph representation of the brain network by considering a threshold *T*.

Phase locking value.

We used the PLV to measure the long-term synchronous changes in neural activity. It was computed as follows:


$$PLV_{i,j}\left(f,w\right)=\frac{1}{f\tau }\left|\overset{f\tau }{\underset{t=1}{\sum }}e^{-i\left(\varphi_{wi}\left(f,t\right)-\varphi_{wj}\left(f,t\right)\right)}\right|$$

where $\varphi_{wi}\left(f,t\right)$ and $\varphi_{wj}\left(f,t\right)$ are wavelet-decomposed time-frequency representations of the difference between the instantaneous phase of the two pairs of channel nodes *i* and *j* at time *t*.

## Brain network analysis

For this analysis, we mainly focused on the efficiencies on two scales: (1) on a macro-scale, the global efficiency referred to the entire network; (2) on a micro-scale, the nodal efficiency measured the mean efficiency of a single network node.

### Macro-scale: Global efficiency

The global efficiency is a measure used to assess parallel exchanging and integrated information processing in a functional brain network. It is calculated as follows:


$$E_{global}\left(G\right)=\frac{1}{N\left(N-1\right)}\underset{j\neq k\in V}{\sum }\frac{1}{d_{j,k}}$$

where *d_j,k_* is the shortest path length between nodes *j* and *k*. For an unweighted binary graph, *d_j,k_* is equal to infinity if there is no path between *j* and *k*; otherwise, it equals the minimum number of edges from *j* to *k*. The global efficiency ranges from 0 to 1, and a higher global efficiency means a higher network capability of performing parallel exchanging or integrated information processing.

### Micro-scale: Nodal efficiency

The nodal efficiency is a measure of the integration of a given node with all other nodes. Given a node *j*, its nodal efficiency is defined as:


$$E_{nodal}\left(j\right)=\frac{1}{\left(N-1\right)}\underset{k\neq j\in V}{\sum }\frac{1}{d_{j,k}}$$

where *d_j,k_* is the shortest path length between nodes *j* and *k*. The nodal efficiency ranges from 0 to 1, and a higher nodal efficiency reflects a higher ability for information transmission or integrated processing with other nodes.

### Feature selection and classification

Based on our hypothesis that MEs and NEs may be associated with different brain network patterns, we aimed to distinguish the brain activities associated with MEs and NEs. We used the functional connectivity matrices of the alpha, beta, and gamma bands as classification features. Furthermore, we divided every 1-s segment of EEG recordings into smaller segments using a 400-ms sliding window with 50% overlap and constructed functional connectivity matrices for each segment.

#### Feature selection

The features were selected using a random forest model. Specifically, the Gini importance of each feature was calculated while fitting the random forest. The Gini importance ranges from 0 to 1, with a larger value representing a more important feature in the classification. We split the dataset into a training set (80% of data) and a test set (20%). The subset of features selected in the training set was reused to train the classification model; ten-fold cross-validation was used to select the optimal feature subset, and the model performance was evaluated using the test set.

#### Brain state classification

A support vector machine (SVM) classifier was used to classify the brain states of the MEs and NEs. Based on the Gini importance, we selected the top 5, 10, 15, 20, 25, and 30 features to fit the classifier. Each time the features were selected, we retrained the classifier using the feature subset of the training set and evaluated the model performance using the test set.

### Statistical tests

For the global efficiency on the macro-scale, Welch’s *t*-test was used to measure the group difference, and a value of *p* < 0.05 was considered statistically significant. For the micro-scale analysis, we first calculated the nodal efficiency for each node. Then, we divided the brain into four regions to lower the influence of multiple comparisons and then measured the differences between regional efficiencies using Welch’s *t*-test, with the false discovery rate (FDR) correction for a low proportion of false positives. Each regional efficiency was calculated by averaging all the nodal efficiencies within the region, and a FDR-corrected value of *p* < 0.05 was considered statistically significant.

## Results

### Macro-scale: Global efficiency

We observed similar global efficiency in the alpha, beta, and gamma bands; in the MEs, it was significantly higher than in the NEs, with *p*-values <0.01 at thresholds of 0.64 and 0.67 in the alpha band, and ≤0.002 at all thresholds in the beta and gamma bands (Table 1). It should be noted from Table 1 that, as the threshold increased from 0.64 to 0.76, the edges of the network became sparse, resulting in a decrease in global efficiency.

**Table 1:** Differences in global efficiency in the alpha, beta, and gamma bands.

Threshold	Alpha			Beta			Gamma		
	MEs	NEs	*p-*value	MEs	NEs	*p*-value	MEs	NEs	*p*-value
0.64	0.26 (0.11)	0.21 (0.12)	<0.001	0.21 (0.10)	0.15 (0.07)	<0.001	0.18 (0.11)	0.15 (0.07)	0.002
0.67	0.21 (0.10)	0.18 (0.11)	0.007	0.17 (0.09)	0.12 (0.05)	<0.001	0.15 (0.10)	0.12 (0.05)	0.001
0.70	0.17 (0.09)	0.15 (0.10)	0.077	0.14 (0.08)	0.10 (0.04)	<0.001	0.13 (0.08)	0.10 (0.04)	<0.001
0.73	0.14 (0.08)	0.13 (0.07)	0.236	0.12 (0.06)	0.09 (0.03)	<0.001	0.11 (0.07)	0.09 (0.03)	<0.001
0.76	0.12 (0.06)	0.11 (0.16)	0.422	0.11 (0.05)	0.08 (0.03)	<0.001	0.10 (0.07)	0.08 (0.03)	<0.001

The values are shown as mean (SD).

### Micro-scale: Nodal efficiency

**Figure 2:**
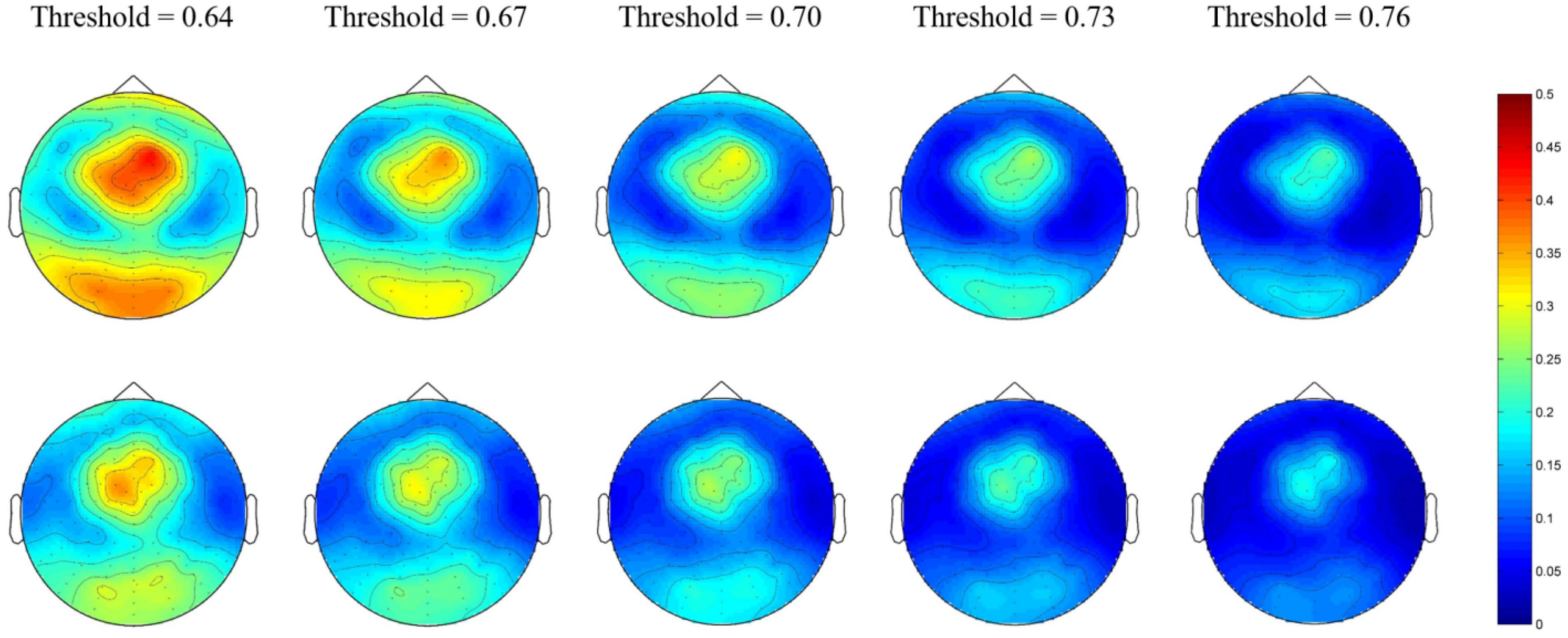
Network topological graphs of the nodal efficiencies in the alpha network. The first row represents the MEs, and the second row the NEs.

At the micro-scale, we first calculated the group-averaged nodal efficiency for each network node. Thereafter, we divided the 128 nodes into four regions (as shown in Figure 1) and calculated each regional efficiency by averaging all the nodal efficiencies in the same region. For the alpha network, the averaged nodal efficiencies are shown in Figure 2. The nodal efficiency in the MEs was significantly higher than in the NEs in the frontal, occipital, and temporal regions at all thresholds, and in the parietal region at thresholds of 0.64 (Figure 2; Table 2).

**Table 2:** Differences in nodal efficiency in the alpha band.

Threshold	Frontal region			Parietal region			Occipital region			Temporal region		
	MEs	NEs	*p*-value	MEs	NEs	*p*-value	MEs	NEs	*p*-value	MEs	NEs	*p*-value
0.64	0.27 (0.12)	0.21 (0.13)	0.002	0.22 (0.08)	0.19 (0.12)	0.009	0.31 (0.16)	0.24 (0.15)	0.002	0.17 (0.08)	0.11 (0.08)	<0.001
0.67	0.21 (0.11)	0.17 (0.11)	0.002	0.17 (0.07)	0.15 (0.11)	ns	0.25 (0.15)	0.19 (0.14)	0.008	0.12 (0.07)	0.08 (0.07)	<0.001
0.70	0.17 (0.09)	0.14 (0.11)	0.010	0.13 (0.06)	0.12 (0.08)	ns	0.21 (0.13)	0.16 (0.12)	0.005	0.09 (0.06)	0.05 (0.05)	<0.001
0.73	0.13 (0.08)	0.11 (0.08)	0.022	0.11 (0.05)	0.09 (0.07)	ns	0.16 (0.12)	0.13 (0.11)	0.009	0.06 (0.05)	0.04 (0.04)	<0.001
0.76	0.11 (0.07)	0.09 (0.07)	0.033	0.07 (0.04)	0.07 (0.06)	ns	0.13 (0.11)	0.11 (0.09)	0.012	0.04 (0.04)	0.02 (0.03)	<0.001

The values are shown as mean (SD). ns, non-significant.

**Figure 3:**
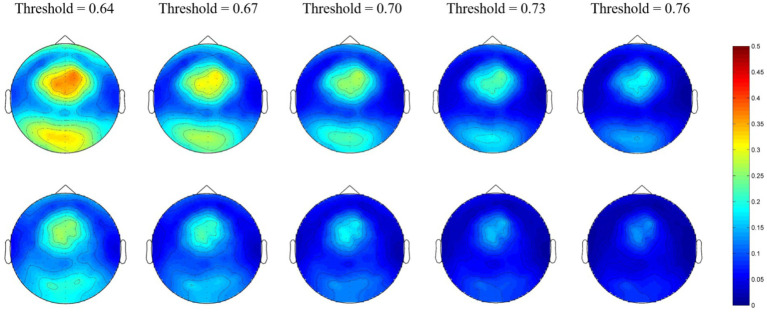
Network topological graphs of the nodal efficiencies in the beta network. The first row represents the MEs, and the second row the NEs.

For the beta network, the averaged nodal efficiencies are shown in Figure 3. The nodal efficiency in the frontal, parietal, temporal, and occipital regions in the MEs was significantly higher than in the NEs at all thresholds (Table 3).

**Table 3:** Differences in nodal efficiency in the beta band.

Threshold	Frontal region			Parietal region			Occipital region			Temporal region		
	MEs	NEs	*p*-value	MEs	NEs	*p*-value	MEs	NEs	*p*-value	MEs	NEs	*p*-value
0.64	0.22 (0.12)	0.15 (0.08)	<0.001	0.18 (0.08)	0.13 (0.07)	<0.001	0.25 (0.15)	0.17 (0.09)	<0.001	0.09 (0.06)	0.06 (0.04)	<0.001
0.67	0.17 (0.11)	0.11 (0.06)	<0.001	0.14 (0.07)	0.11 (0.05)	<0.001	0.21 (0.13)	0.13 (0.07)	<0.001	0.06 (0.05)	0.04 (0.03)	<0.001
0.70	0.14 (0.09)	0.08 (0.05)	<0.001	0.11 (0.06)	0.08 (0.04)	<0.001	0.16 (0.11)	0.11 (0.06)	<0.001	0.04 (0.04)	0.03 (0.02)	0.002
0.73	0.11 (0.08)	0.06 (0.04)	<0.001	0.08 (0.05)	0.06 (0.03)	<0.001	0.13 (0.09)	0.07 (0.04)	<0.001	0.03 (0.03)	0.02 (0.02)	0.002
0.76	0.09 (0.06)	0.05 (0.03)	<0.001	0.06 (0.04)	0.04 (0.02)	<0.001	0.11 (0.08)	0.06 (0.03)	<0.001	0.02 (0.03)	0.01 (0.01)	0.008

The values are shown as mean (SD).

**Figure 4:**
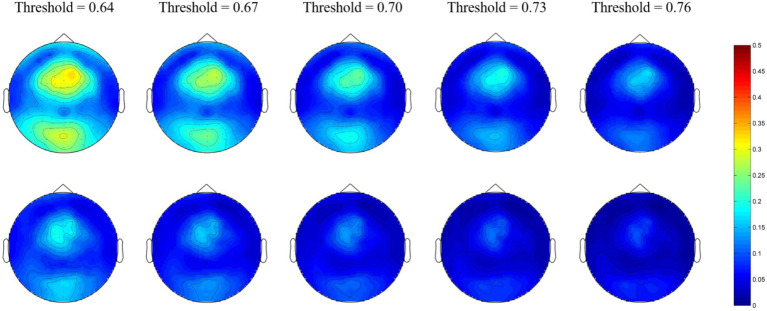
Network topological graphs of the nodal efficiencies in the gamma network. The first row represents the MEs, and the second row the NEs.

For the gamma network, the averaged nodal efficiencies are shown in Figure 4. The nodal efficiency of the frontal, parietal, and occipital regions in the MEs was significantly higher than in the NEs at all thresholds (Table 4). However, there was no significant difference in nodal efficiency in the temporal region at a threshold of 0.76.

**Table 4:** Differences in nodal efficiency in the gamma band.

Threshold	Frontal region			Parietal region			Occipital region			Temporal region		
	MEs	NEs	*p*-value	MEs	NEs	*p*-value	MEs	NEs	*p*-value	MEs	NEs	*p*-value
0.64	0.19 (0.13)	0.10 (0.06)	<0.001	0.16 (0.09)	0.10 (0.06)	<0.001	0.22 (0.15)	0.13 (0.07)	<0.001	0.08 (0.08)	0.06 (0.05)	<0.001
0.67	0.16 (0.12)	0.08 (0.05)	<0.001	0.12 (0.08)	0.07 (0.05)	<0.001	0.17 (0.13)	0.10 (0.06)	<0.001	0.06 (0.08)	0.04 (0.04)	0.003
0.70	0.12 (0.10)	0.06 (0.04)	<0.001	0.10 (0.07)	0.06 (0.04)	<0.001	0.14 (0.12)	0.08 (0.05)	<0.001	0.05 (0.07)	0.03 (0.03)	0.015
0.73	0.10 (0.09)	0.05 (0.03)	<0.001	0.07 (0.06)	0.04 (0.03)	<0.001	0.11 (0.10)	0.07 (0.04)	<0.001	0.03 (0.06)	0.02 (0.02)	0.037
0.76	0.08 (0.08)	0.04 (0.03)	<0.001	0.06 (0.05)	0.03 (0.03)	<0.001	0.09 (0.09)	0.06 (0.03)	0.001	0.03 (0.05)	0.02 (0.02)	ns

The values are shown as mean (SD). ns, non-significant.

### Feature selection and classification

**Figure 5:**
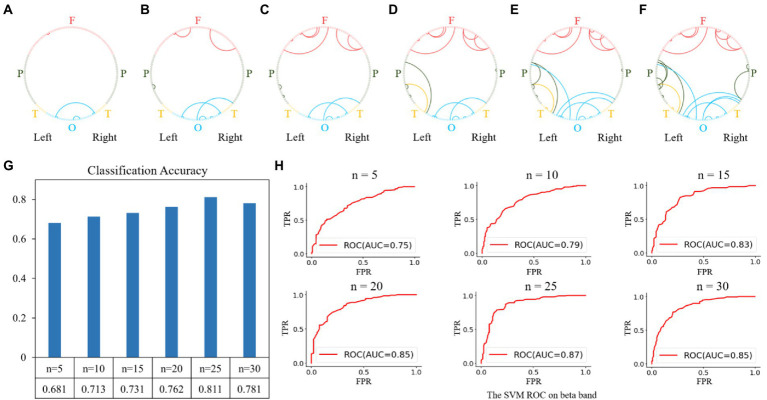
Feature selection and classification accuracy on the beta band. **(A–F)**: Top n features (from 5 to 30 with a step size of 5); **(G,H)**: Classification accuracies and receiver operating characteristic (ROC) curves with SVM classifier using the top n features. The highest classification accuracy was 0.811 with an AUC of 0.87, obtained using the top-25 features.

Feature selection and brain state classification were performed based on the functional connectivity matrices of the alpha, beta, and gamma bands. Since the beta band classification accuracy was higher, we used that band as a sample to illustrate both the feature selection and classification procedures. According to the Gini importance, we sorted the features in ascending order and selected the top-n features (from 5 to 30, with a step size of 5) to classify the different brain states between MEs and NEs. The top-5 features were the four functional connectivities within the occipital and frontal regions, as shown in Figure 5A, which were previously used as classification features. With the selected top-5 features, we fitted an SVM classifier on the training set and then measured its generalization performance using the test set with ten-fold cross-validation. The prediction accuracy obtained on the test dataset was 0.681, with an area under the receiver operating characteristic curve (AUC) of 0.75, as shown in Figures 5G,H. Subsequently, we expanded the features from the top 5 to the top 10. The top-10 features were the functional connectivities that increased in the channels in the frontal and parietal regions, as shown in Figure 5B. The prediction accuracy obtained on the test dataset increased to 0.713 with an AUC of 0.79. Each time the number of features increased, we retrained the classifier using the training set and measured its generalization performance with the test set with 10-fold cross-validation. The top 15, 20, 25, and 30 features are shown in Figures 5C–F, and the classification performances are shown in Figures 5G,H. Using the top 25 features, we achieved a relatively high classification accuracy of 0.811 with an AUC of 0.87.

**Figure 6:**
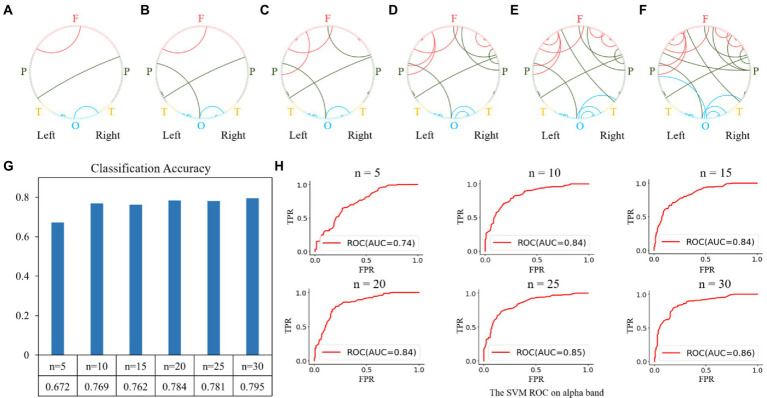
Feature selection and classification accuracy on the alpha band. **(A–F)**: The top n features (from 5 to 30 with a step size of 5); **(G,H)**: Classification accuracies and receiver operating characteristic (ROC) curves with SVM classifier using the top n features. The highest classification accuracy was 0.795 with an AUC of 0.86, obtained using the top-30 features.

Similar feature selection and classification processes were performed using the functional connectivity matrices in the alpha and gamma bands as the classification features. The results of the feature selection on the alpha band are shown in Figure 6. The top-5 features were the functional connectivities within the parietal, occipital, and frontal regions, as shown in Figure 6A. The top 10, 15, 20, 25, and 30 features are shown in Figures 6B–F, and the corresponding classification performances are shown in Figures 6G,H. We obtained a relatively high classification accuracy of 0.795 with an AUC of 0.86, using the top 30 features.

**Figure 7:**
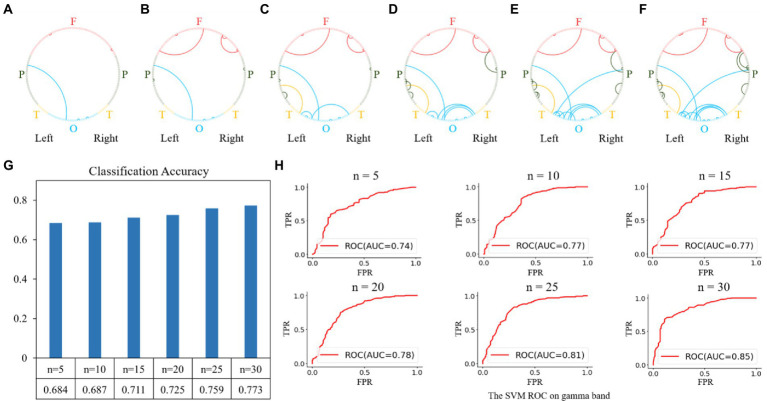
Feature selection and classification accuracy on the gamma band. **(A–F)**: The top n features (from 5 to 30 with a step size of 5) on the gamma band; **(G,H)**: Classification accuracies and receiver operating characteristic (ROC) curves with SVM classifier using the top n features. The highest classification accuracy was 0.773 with an AUC of 0.85, obtained using the top-30 features.

For the gamma band, the results of the feature selection are shown in Figure 7. The top-5 features were the functional connectivities within the parietal, occipital, and frontal regions, as shown in Figure 7A. The top 10, 15, 20, 25, and 30 features are shown in Figures 7B–F, and the corresponding classification performances are shown in Figures 7G,H. The highest classification accuracy for the gamma band was 0.773 with an AUC of 0.85, obtained using the top 30 features.

## Discussion

This study uses the SEES paradigm and EEG to measure the brain network changes during ME occurrence, exploring the brain mechanisms of ME from a neural perspective for the first time. Additionally, we used complex network analysis and machine learning algorithms to distinguish the functional connectivity between MEs and NEs and provide electrophysiological indicators for ME recognition. The main findings of our study are as follows: (1) on the macro-scale, the global efficiency related to MEs was higher than that related to NEs in the alpha, beta, and gamma bands; (2) on the micro-scale, the nodal efficiencies related to MEs were higher than those of NEs in the frontal, temporal and occipital regions; (3) the classification accuracy of the SVM reached 0.81 with an AUC of 0.85 in a beta band based on the difference between MEs and NEs in the brain network with only a small number of functional connection features (*N* = 25) selected using the random forest algorithm.

The MEs were related to higher global efficiency in the alpha, beta, and gamma networks compared with NEs. Previous research has found that a higher global efficiency is associated with greater cognitive load (Kitzbichler et al., 2011; Yu et al., 2021). For example, Kitzbichler et al. (2011), using the N-back task, found that the cognitive load of the participants increased in relation to the increased difficulty of the task and the higher global efficiency of the brain network (Kitzbichler et al., 2011). Yu et al. (2021) also found that the brain is higher globally efficient when processing 3D videos with a greater cognitive load than 2D videos as they require the processing of more information, such as the depth structure (Yu et al., 2021). Therefore, the generation of MEs may require a greater cognitive load than the NEs, as shown by higher global efficiency. Frank and Svetieva (2015) indicated that the generation of MEs involves a dual system of expression processing (the interaction of the pyramidal and extrapyramidal motor systems) and also involves emotion arousal and inhibitory processes (Frank and Svetieva, 2015). It is not surprising that these complex tasks require a greater cognitive load and higher global efficiency.

Combining the topographic and difference analyzes of nodal efficiency, we found that participants showed higher efficiency in micro-expressions in both alpha, beta, and gamma networks, mainly in the frontal, temporal, and occipital regions. This finding may be related to the fact that MEs production involves higher emotional arousal and stronger cognitive control than NEs (Bartholomew et al., 2019, 2021). Emotional arousal relies on the emotional network, which mainly includes the occipital and temporal lobes, and increased network efficiency in occipital and temporal lobes may be associated with increased intensity of emotional arousal (Dolan, 2002; Bartholomew et al., 2021; Zhuang et al., 2021). Previous studies have found that an increased network efficiency of the emotional network correlates with the intensity of emotional arousal (Zhang et al., 2015). Comparative studies also found that positive emotional stimuli induced higher network efficiency compared with neutral stimuli (Sun et al., 2020). In general, emotion-related cognitive control mainly involves the frontal regions, and increased network efficiency in these regions is often associated with an increased requirement for cognitive control (Bartholomew et al., 2021). For instance, in classical tasks, such as the color-word Stroop, high inhibitory demand in the inconsistent condition increased the efficiency of the frontal area network (Bartholomew et al., 2021). Another study using the emotion-word Stroop task found that emotional inhibition is associated with increased efficiency in the frontal region (Bartholomew et al., 2021). Thus, the higher efficiency related to MEs in the occipital, temporal, and frontal lobes compared with NEs may be associated with higher emotional arousal levels and stronger cognitive control processing.

In this study, different brain activities associated with MEs and NEs were classified according to the functional connectivity matrix of the alpha, beta, and gamma bands. We used a random forest-based feature selection model to identify key features with high Gini importance to improve the classification performance and avoid possible overfitting. Random forest is an integrated machine learning algorithm based on bootstrap aggregation. Random forest is suitable for high-dimensional feature spaces, such as in bioinformatics, thanks to their ease of computation and parallelization. Using selected features, we successfully classified different brain activities using an SVM classifier. With only a small number of functional connection features (*N* = 25) selected using the random forest, the SVM achieved a classification accuracy of 0.81 and an AUC of 0.85. This study provides the first electrophysiological metrics for ME recognition from a neuroscience perspective. This result illustrates the possibility of using physiological measures for ME identification instead of image recognition, with a wider range of application scenarios, such as for persons wearing face masks, or in low visibility environments, or for patients with expression disorders.

Only MEs arising with a positive emotion were examined in this study because Johnston et al. argue that smiling requires little preparation, and smiling is usually used more frequently than other emotional expressions (Johnston et al., 2010). Also, in social interactions, smiling is usually encouraging and has a communicative function; therefore, MEs under positive emotions are more common. Future research should examine the brain mechanisms related to MEs under negative emotions.

## Conclusion

This study explores for the first time the brain mechanisms underlying micro-expressions. Compared with the NEs, occurrence of MEs is characterized by higher global efficiency, and higher nodal efficiency in the frontal, occipital, and temporal regions. It demonstrated that MEs had a greater cognitive load compared with the NEs, associated with higher emotional arousal levels and stronger cognitive control processing. Our SVM classifier based on EEG signals achieved a classification accuracy for MEs and NEs of 0.81 with an AUC of 0.85. This result demonstrated the possibility of using EEG to recognize MEs, which could have a wider range of application scenarios than image recognition, such as for persons wearing face masks or patients with expression disorders.

## Data availability statement

The data that support the findings of this study are available from the corresponding author, upon reasonable request.

## Ethics statement

The studies involving human participants were reviewed and approved by ethics committee of Southwest University. The patients/participants provided their written informed consent to participate in this study.

## Author contributions

XZh, XZe, and SW performed the formal analysis. XZh wrote the original draft of the manuscript. JC, YL, TC, and GL reviewed and edited the manuscript. All authors contributed to the article and approved the submitted version.

## Funding

This work was supported in part by the National Natural Science Foundation of China (grant nos. 61472330 and 61872301).

## Conflict of interest

The authors declare that the research was conducted in the absence of any commercial or financial relationships that could be construed as a potential conflict of interest.

## Publisher’s note

All claims expressed in this article are solely those of the authors and do not necessarily represent those of their affiliated organizations, or those of the publisher, the editors and the reviewers. Any product that may be evaluated in this article, or claim that may be made by its manufacturer, is not guaranteed or endorsed by the publisher.
